# Reactive mass vaccination campaign against cholera in the COVID-19 context in Cameroon: challenges, best practices and lessons learned

**DOI:** 10.11604/pamj.2021.38.392.27754

**Published:** 2021-04-22

**Authors:** Adidja Amani, André Arsène Bita Fouda, Armanda Jeanne Nangmo, Solange Ngo Bama, Collins Asaah Tatang, Marie Angèle Mbang, Emmanuel Christian Epee Douba, Aimé Gilbert Mbonda Noula, Mariette Dia, Malika Bouhenia

**Affiliations:** 1 Sub-Directorate of Vaccination, Directorate of Family Health, Ministry of Public Health, Yaoundé, Cameroon,; 2 World Health Organization, Regional Office for Africa, Vaccine-Preventable Disease Unit, Brazzaville, Republic of Congo,; 3 Evodoula District Hospital, Evodoula, Cameroon,; 4 Muea Medicalized Center, Buea, Cameroon,; 5 National Operating Office, Disease Prevention and Control Office, Emergency Preparedness and Control Office, World Health Organization Country Office, Yaoundé, Cameroon,; 6 Community Epidemic and Pandemic Preparedness, International Federation of Red Cross and Red Crescent Societies Cluster Support Team, Central Africa Region, Yaoundé, Cameroon,; 7 World Health Organization Africa Office, Brazzaville, Republic of Congo,; 8 World Health Organization Main Office, Geneva, Switzerland

**Keywords:** Challenges, practices, lessons learned, cholera, vaccination, Cameroon, COVID-19

## Abstract

**Introduction:**

since 1971, Cameroon is facing a growing series of cholera epidemics despite all the efforts made by the government to address this huge public health threat. In 2020, in addition to the COVID-19 pandemic, Cameroon recorded a high cholera case fatality rate of 4.3% following epidemics noted in the South, Littoral and South-West regions. The Cameroon Ministry of Public Health, has thus organized a reactive vaccination campaign against cholera to address the high mortality rate in the affected health districts of those regions. The objective of this study was to describe the challenges, best practices and lessons learned drawing from daily experiences from this reactive vaccination campaign against cholera.

**Methods:**

we conducted a cross-sectional study drawn from the results of the campaign. We had a target population of 631,109 participants aged 1 year and above resident of the targeted health areas.

**Results:**

the overall vaccination coverage was 64.4% with a refusal rate ranging from 0-10% according to health districts. Vaccination coverage was the lowest among people aged 20 years and above. The main challenge was difficulty maintaining physical distanciation, the main best practice was the screening of all actors taking part at the vaccination against COVID-19 and we found that emphasizing on thorough population sensitization through quarter heads and social mobilizers and adequately programming the campaign during a good climate season is crucial to achieving good vaccination coverage.

**Conclusion:**

lessons learned from this study could serve to inform various agencies in the event of planning rapid mass vaccination programs during pandemics.

## Introduction

Cholera is a human diarrheal disease endemic in Africa, caused by the toxins of the bacteria called *Vibrio cholera* [1-3]. It is manifested by watery diarrhea with a rice-white appearance emitting a peculiar odor which dehydrates the patient and is lethal if proper care is not provided promptly [4,5]. This disease continues to wreak havoc in poor countries with limited resources, including Cameroon, where access to drinking water remains a real and growing challenge for some populations.

The spread of cholera is dependent on numerous environmental and biological variables, including seasonal environmental drivers, host immunity, the infectivity of the bacteria and lytic bacteriophages [6]. According to Andrew Azman´s systematic review that was carried out in 2013, toxigenic cholera median incubation period is 1.4 days with 5% of cases symptoms by 0.5 days and 95% by 4.4 days [7]. Cholera epidemics are expected to kick off during episodes of prevailing warm air temperature with stagnant river flows, creating favorable environmental conditions for the growth of cholera bacteria [8]. Heavy rainfall, through floods and/or breakdown of sanitary infrastructures, accelerates interaction between contaminated water and human activities, resulting in an epidemic [8]. Also, in some contexts like Cameroon, there are risk factors like extreme poverty and promiscuity where transmission occurs through foods, water and close contact which favour an epidemic to set in [1].

Despite inadequate cholera cases reporting due to surveillance systems failings [9-11], in 2015, the global burden of cholera was estimated at 2.86 million (ranging from 1.4 to 4 million) of cases resulting in 95000 deaths (with significant differences in estimates ranging from 21,000 to 143,000) [10]. Even though all regions of the world are affected by cholera, in 2014, sub-Saharan Africa (SSA) remained the most affected continent with approximately 90% of cholera cases [12]. In 2019, 13 African countries reported a total of 55,087 cases and 872 deaths, corresponding to a case fatality rate (CFR) of 1.6% with Cameroon bearing the highest CFR [13]. Cameroon reported its worst outbreak in 2011 (22,762 suspected cases and 786 deaths) [14] since the first case of cholera was registered on February 4, 1971 [15,16]. The annual case fatality rates are exhibiting steep upward trend and remain consistently well above the acceptable World Health Organization (WHO) value of 1%. In 2018, Cameroon had 997 suspected cases and 58 deaths yielding a CFR of 5.8% [17]. In 2019, 478 suspected cases and 19 deaths (CFR of 4.0%) [18] and during the 40^th^ epidemiological week of 2020, there were 1,848 suspected cases and 80 deaths yielding a CFR of 4.3%.

Based on the cholera risk assessment conducted by the WHO in 2018 in Cameroon, these regions fulfil all the necessary conditions for a reactive vaccination campaign. First, some health areas in these regions face a serious lack of safe water and poor hygienic conditions, as well as poor access to health care services. Second, as the South-West region has suffered a socio-political crisis for four consecutive years, life insecurity another prominent factor. Third, the Londji Health Area (South region) comprises mainly islets with no access to latrines and its population consists primarily of fishermen. This area is also characterized by a large influx of surrounding population in need of fish. Thus, poor hygiene coupled with unfavourable living conditions, poor drinking water coverage and life insecurity pose a serious risk to the local population. Moreover, owing to the contiguity between the Littoral and the South-West region, residents of these three regions are particularly vulnerable to the spreading of a cholera epidemic.

Since the beginning of COVID-19 pandemic, the WHO has issued guidelines indicating that mass vaccination campaigns should cease until the COVID-19 situation resolves [19,20]. However, given the sanitary risks to which some Cameroonians localities are exposed and considering the decision-making framework for the implementation of mass vaccination campaigns in the context of COVID-19 [21], a reactive mass vaccination campaign against cholera was deemed necessary in the Littoral, South and South-West regions. The organization of this campaign is part of an integrated approach, complementary to contributing to ending cholera global 2030 roadmap strategy [22] and to all other cholera interventions implemented in the COVID-19 context by the ministry of public health, in collaboration with technical and financial partners.

Besides, even though prompted by the current COVID-19 situation in the world, the WHO and the Cameroon Ministry of Public Health prescribed hygienic measures and restricted movement, the resurgence of cholera outbreaks represents a double dividend of health risks to certain populations in Cameroon. Against this backdrop, the objective of this article is to describe vaccination coverage and discuss challenges, lessons learned and best practices for carrying out a mass vaccination campaign against cholera during COVID-19 with the overarching goal of informing global health organizations and leaders on the campaign outcomes.

## Methods

**Study design:** this was a cross-sectional study employing the results of the reactive mass vaccination campaign against cholera carried out from August to September 2020. It was conducted in some health areas (HAs) of cholera affected health districts (HDs) of the Littoral, South and South-West regions of Cameroon which recorded at least one cholera case from March to August 2020.

**Study setting:** the study was conducted in the following HDs and HAs. In the Littoral region, we had the HD of Bonassama (Mabanda and Bonassama HAs), New-Bell (Nkolouloun, Sebenjongo, Mbam Ewondo, New-Bell Bamiléké, Makea and Youpwe HAs), Nylon (Soboum) and Japoma (Bwang). In the South region, we had the Kribi HD (Londji, Adjap, Grand-Batanga, Hevecam and Kribi HAs). In the South-West region Limbe HD (Mabeta HA) and Tiko HD (Holforth, Kange, Likomba, Missellele, Mondoni, Mudeka, Mutegene, Tiko Town HAs). It is worth noting that, a vaccination campaign was also carried out in Manoka HD (Littoral region) in February 2020 but these data are not included in this paper as it was not carried out during the COVID-19 pandemic in Cameroon.

**Study target population:** this study was conducted among people of both sexes, aged one year and above, including pregnant women. Health areas target population was drawn from the Cameroon Ministry of Public Health 2020 target populations (Table 1).

**Table 1:** study target population for the oral cholera vaccines (OCV) in the three regions in August-September 2020

Variables	Participants (n=154)	Percentage (%)
**Age Groups**		
18 - 30	27	17.5
31 - 40	16	10.4
41 - 50	23	14.9
51 - 60	38	24.7
61 - 70	35	22.7
71 - 80 81 - 90	10 5	6.5 3.2
Mean ± SD	51.2 ± 16.8	
Range	18 - 88	
**Gender**		
Male	71	46.1
Female	83	53.9
**Duration of symptoms (Months)**		
3 - 24	74	48.1
25 - 48	43	27.9
49 - 72	21	13.6
Above 72	16	10.4
Mean ± SD	43.9 ± 38.2	
Range	3 - 240	

**Leadership and planning:** to carry out this vaccination campaign, a preparation plan was put in place. Preparatory meetings at the central and regional level were held after approval by the Cameroon Ministry of Public Health. The WHO has declared its intention to fund the operational costs of the campaign. At the regional level, the following activities were conducted: advocacy with the administration, traditional and religious authorities; raising awareness of worshippers in places of prayer, messages to key sectors and community organizations; production of sensitization materials such as banners and posters; briefing meetings between the public health regional delegates and their staff, central supervisors, district executive teams and partners on the organization of the campaign. A coordinating committee at the regional level and in the Health districts was put together.

**Oral cholera vaccines (OCV) procurement:** Shanchol™ and Euvichol-Plus^R^ were the two bivalent OCV used during this campaign. A total of 1,168,996 doses of Schanchol™ and Euvichol-Plus^R^ were made available by the International Coordinating Group (ICG) emergency stockpile. The WHO facilitated the availability of operational costs for the campaign. Besides, doctors without borders helped with logistics in Kribi HD. Shanchol™ was used in the Littoral region and Euvichol-Plus^R^ in the South and South-West regions.

**Cold chain and waste management:** vaccines were transported from the central cold room to the 3 regional cold rooms because many of these HDs/HAs did not have an adequate cold chain system. Arrangements were made for adequate storage of vaccines at the local level between +2°C to +8°C until they were administered as recommended by the manufacturers.

**Staff training:** after briefing the district senior teams at the public health regional delegations, social mobilizers and vaccinators were trained. The training was conducted by pools with the support of central supervisors and WHO partners to respect physical distanciation. In total, 2271 people were trained for the campaign; 1482 persons, 489 persons and 300 persons in the Littoral, South-West and South regions respectively.

**COVID-19 screening:** before being enrolled as a social mobilizer or a vaccinator, each volunteer had to be screened for COVID-19 using the Rapid Diagnostic Test SD BIOSENSOR COVID-19 Ag to avoid infecting others during the vaccination campaign against cholera.

**Data analysis:** data were collected through vaccination tally sheets. The treatment and analysis of data was done through Excel version 2013.

## Results

**Social mobilization:** as part of community sensitization, the objective was to inform at least 95% of household´s heads before the visit of vaccination teams.

**Littoral region:** we can observe that refusal cases were so many in the Littoral region (Table 2). The average was 7% ranging from 3% in the Nylon Health District to 10.28% in the Japoma Health District. The number of refusal cases we were not able to manage in this region was also high. In the Littoral region, the main refusal reasons were “undetermined causes” (66%) followed by “not being the person who decides” (13%) and then a dangerous vaccine (11%). According to the independent monitoring carried out by WHO partners the main information pathway was through social mobilization (71%).

**Table 2:** OCV social mobilization results in the three regions

Region	Health district	Number of visited households	Number of people sensitized	Number of refusals	Number of refusals managed	Number of refusals not managed
Littoral	Bonassama	20,248	46,813	3,472	224	3,248
	New-Bell	24,216	53,088	3,807	519	3,288
	Nylon	6,586	14,333	448	99	349
	Japoma	2,224	4,628	476	54	422
	Total	53,274	118,862	8,203	896	7,307
South-West	Limbe	1,318	3,714	-	-	-
	Tiko	37,109	155,179	-	-	-
	Total	38,427	158,893	-	-	-
South	Londji	1852	4070	57	15	42
	Adjap	450	2109	4	3	1
	Hevecam	1369	4449	20	2	18
	Grand-Batanga	901	3875	17	2	15
	Kribi		2000	98	10	88
	Total		16503	196	32	164

OCV: oral cholera vaccine

**South-West region:** in the South-West region for instance, the main refusal causes were “being completely vaccinated” (51%) and “undetermined causes” (46%). The main pathway of information was griots (43%) followed by social mobilization (37%).

**South region:** the refusal rate in the South region is 1.2%. The main population information pathway was through social mobilization (26%) followed by the health personnel (19%).

### Administrative vaccine coverage

**Figure 1:**
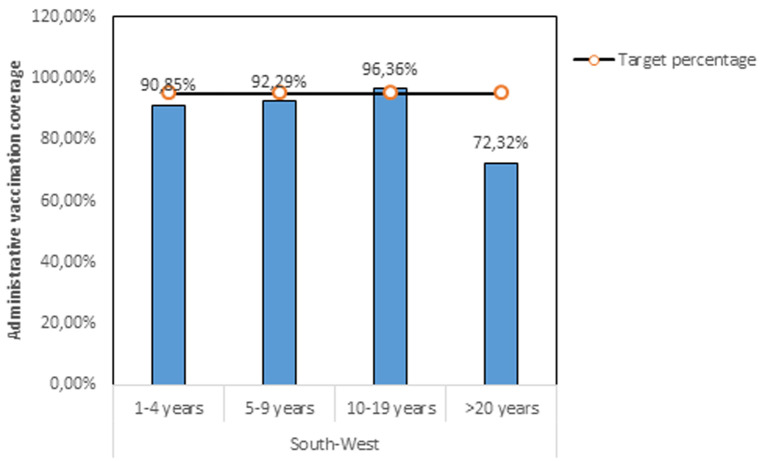
vaccination coverage rate according to age ranges during the OCV campaign in the South-West region of Cameroon

**South-West region:** these results reveal the different oral cholera vaccination coverage in the affected health areas of the South-West region (Table 3). The overall vaccination coverage in the South-West region was 81.97% but greatly varying across age ranges and health areas. Only two health areas in the South-West region achieved an acceptable vaccination coverage >95%. There was a high vaccination coverage in Mabeta (Limbe Health District) which is a small health area of 6899 people because of ease of geographic access through roads as compared to health areas of Tiko District where you have to take canoe sometimes to reach certain health areas. Besides, in Limbe, they were seeing fresh cases of cholera dying around them, this also increased their fear of the disease and vaccine acceptance. In Figure 1, we can observe there was less than 80% vaccination coverage among populations aged 20 years and above. This might be due to the belief that vaccination is linked to children with some parents vaccinating their children and don´t get vaccinated themselves. Only the adolescent´s population achieved the target vaccination rate of at least 95%.

**Table 3:** OCV vaccination coverage in the three regions

Region	Health areas/health districts	2020 total population	2020 target population >1 year at risk of cholera	Persons vaccinated	Administrative coverage
South-west	Mabeta/Limbe	7,098	6,899	6,645	96,32%a
	Holtforth	38,864	37,698	20,596	54,63%c
	Kange	4,831	4,686	5,311	113.33%a
	Likomba	12,246	11,879	8,946	75,31%c
	Missellele	6,505	6,323	5,434	85,94%b
	Mondoni	3,446	3,350	2,695	80,45%b
	Mudeka	10,647	10,349	8,266	79,87%c
	Mutengene	55,653	54,095	52,876	97,75%a
	Tiko town	22,498	21,868	18,045	82,52%b
	Total Limbe/Tiko	161,788	157,147	128,814	81,97%b
Littoral	Bonassama	127,055	124,132	25,064	20,19%c
	New-Bell	192,595	188,166	83,726	44,50%c
	Nylon	46,477	45,408	27,929	61,51%c
	Japoma	8,817	8,614	4,750	55,14%c
	Total	374,944	366,320	141,469	38.61%c
South	Londji	8,130	7,894	7,547	95.60%
	Adjap	4,309	4,184	3,462	82.74%
	Grand Batanga	11,739	11,399	10,039	88.07%
	Hevecam	22,659	22,002	17,978	81.7%
	Kribi	64,020	62,163	39,272	63.18%
	Total	110,857	107,642	78,298	72.74%

a: vaccination coverage >95%; b: vaccination coverage between 80 and 95%; c: vaccination coverage less than 80%

**Littoral region:** the overall coverage rate in the Littoral region was the lowest despite the huge target population. This might be due to the poor perception of cholera risk or to the inherent behaviour (doubtful character) of people living in the Littoral region. Secondly, this campaign was conducted entirely during August when there are torrential rainfalls in the Littoral region with trees falling on roads and preventing the passage of teams or cars. The latter prevented vaccination teams from going into the field. The initial vaccination schedule of 2 days was prolonged for 4 more days. Also according to the independent monitoring that was carried out by WHO, we also had a great number of missed households, with some stating they were not informed of the campaign. In the Littoral region, reasons for non-vaccination varied greatly across health districts. According to the WHO independent monitoring, the main reasons of non-vaccination in the Littoral region were other reasons (37%) followed by travel (31%), being in the market (18%), school (18%) farm and playing outside.

**Figure 2:**
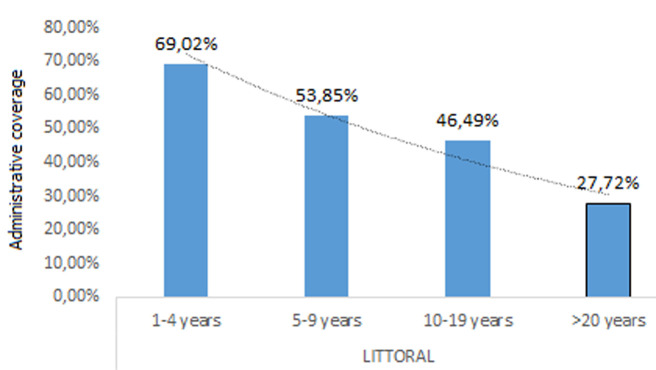
outlines that as age increases the vaccination coverage decreases in the Littoral region

**Figure 3:**
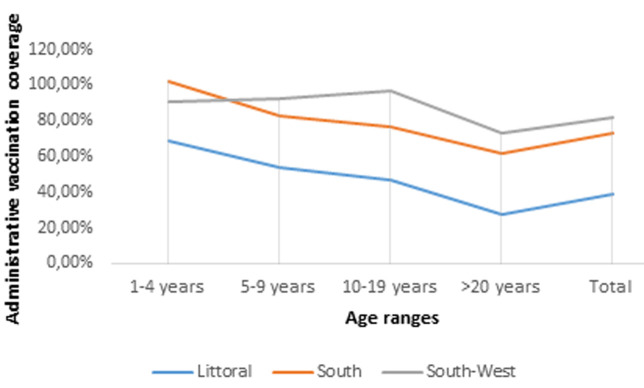
administrative vaccination coverage according to the age ranges in the three regions

**South region:** the overall vaccination coverage was low (Figure 2). In the South region, the campaign started in the Londji health area. The vaccination coverage there was good in Londji because population accepted the vaccination because they had fresh dying cases of cholera in their locality. In other health areas, vaccination coverage was also poor because of the torrential rainfalls. This vaccination overall coverage is 64.43% which is far lower than the acceptable vaccination coverage of 85%. The Littoral and the South region follow the same distribution with children between 1-4 years having the highest coverage and adults >20 years having the lowest coverage rate (Figure 3). The South-West region faces a different distribution pattern with adolescents aged 10-19 years having the greatest vaccination.

**Challenges, best practices, and lessons learned:** WHO has recommended the use of hydro-alcoholic solutions and face masks for mass vaccination campaigns. The term personal protective equipment (PPE) in the following therefore refer to them.

### Challenges

**Difficulty to practice physical distanciation by the population:** even though the predominant vaccination strategy was the door-to-door strategy, we had to set up fixed and temporarily fixed posts in some chiefdoms and health facilities. This favoured people coming together. It was therefore quite impossible to maintain physical distanciation.

**Insufficient supply of hydro-alcoholic solutions and face masks for vaccination staff:** in general, hydro-alcoholic solutions and face masks were distributed by regions according to their target population but the quantity given in some health areas was insufficient for the staff present. PPEs were distributed according to the target population of each health area.

**Difficulty recruiting volunteers especially social mobilizers in the South-West:** it was difficult to recruit volunteers´ especially social mobilizers in the South-West region considering the poor socio-political crisis that is ongoing in the region. People were afraid to wear anything concerning the government as it might put their lives at risk. Also, they were so much concerned about their health in the COVID-19 context.

**High refusal rate among some populations:** vaccine refusal was a great challenge in the Littoral region therefore threatening achievements made so far concerning immunization services. The high refusal rate might be firstly inherent to the Littoral´s population behaviour. People were also hesitant taking OCV thinking this was COVID-19 vaccine that was been administered to them as some teams wore protection gowns which were not recommended.

**Heavy rainfalls:** during the implementation of the campaign, there were heavy rainfalls in the Littoral and South regions which prevented the effective implementation of vaccination. Vaccination was scheduled for six days but teams were able to vaccinate only for two days therefore reducing our vaccination coverage.

**The lack of adequate gadgets to identify vaccination teams:** vaccinators and social mobilizers were not identifiable by their badges and their aprons. This undermined the social mobilization. Worse, wearing the combinations sued for the fight against COVID-19 fueled the rumor that it could be COVID-19 vaccine being administered.

### Best practices

**To carry out the first-ever oral cholera vaccination campaign in COVID-19 context despite restrictions:** while we acknowledge WHO recommendations on differing mass vaccination campaigns until COVID-19 stops, we faced a critical cholera epidemic with several active districts in three regions of Cameroon in 2020. Therefore, organizing a reactive mass vaccination against cholera in those districts was mandatory. This campaign helped in stopping the epidemic in those health areas while preventing the spreading of COVID-19.

**Screening of all actors and vaccination staff against COVID-19 using rapid diagnostic tests:** right before the beginning of the campaign, all the staff involved in the campaign were screened against COVID-19. Above increasing the country testing rate, it also helped to prevent contamination from a staff to a vaccination participant. Fortunately, all of them were COVID-19 free at the moment of testing. Despite testing all actors at the beginning of the campaign, its implementation was not complete because no testing was done at mid-campaign and the end of the campaign. Considering the incubation period of COVID-19 ranges from 5 to 14 days, vaccination teams might have been exposed to either mild or severe COVID-19 cases during the campaign.

**The availability of personal protective equipment´s (PPEs):** despite being insufficient for all vaccination staff, all the health areas involved in the campaign were offered PPEs.

**Training of vaccination staff by pools:** in total, 2244 mobilizers and vaccinators were to be trained in the three regions for the campaign. The Cameroonian government said not more than 50 persons should be present in one room. We looked for schools that had several classes to train vaccination team´s classes after classes to maintain physical distanciation.

### Lessons learned

**An emphasis should be put on sensitization at least 2 weeks before the implementation of the campaign:** during the campaign, we noticed populations were much more reluctant because they reported not being aware of an ongoing cholera vaccination campaign. This will allow a significant reduction in reluctance. The availability of a communication plan with emphasis on the involvement of quarter chiefs allow a deep population sensitization. Providing an incentive budget line for the community leaders tightly related to the population might largely influence the outcome of the vaccination campaign. At the beginning of the campaign, in the Littoral region (New-Bell health district), people were too reluctant to take the oral cholera vaccination, but after holding a meeting with the neighbourhood chiefs, the vaccination coverage steeply increased. So we believe motivating community leaders might have a great impact on the vaccinations coverage. This lesson can also help in educating populations on adopting COVID-19 hygienic measures by greatly involving also financially their community leaders.

**Provision of water points:** in addition to hydro-alcoholic solutions and face masks, we should either provide water points so that vaccinators can wash their hands frequently during a mass vaccination campaign or increase the quantity of hydro-alcoholic solutions.

**Implementation of mass vaccination campaigns should be done in the dry season:** carrying out a mass vaccination campaign in seaside towns might be challenging. From now on, we will organize our vaccination campaign during the dry season. This to permit effective implementation of the campaign and adequate management of resources.

## Discussion

This article describes vaccination coverage, challenges, best practices and lessons learned during the OCV vaccination coverage in the Littoral, South and South-West regions in Cameroon from August to September 2020. We observed a very low vaccination coverage of 64% overall. This coverage is far lower than that obtained in Guinea in 2012 and in South Sudan in 2016 [23,24]. This probably due to the poor climatic conditions during which the campaign was conducted. Unfortunately, this administrative vaccination coverage also greatly varied across regions: in the Littoral region (38.61%) as compared to the South (72.74%) and South-West (81.94%) regions. One of the reasons explaining this great disparity among regions might be the superiorly urban aspect of the Littoral region and the high density of its population. We observe that as a community grow in urbanity, its OCV vaccination coverage tends to decrease. These findings are comparable to what was obtained in Haiti in 2012 where they had 71% in rural Brocozel [25] and 69% in urban slums in Port au Prince [26].

We also observe an overall tendency of very low vaccination coverage among people aged 20 years and above. Similar findings were observed among people 15 years and above in Haiti [27], Mozambique [28], Bangladesh, India [29], Guinea [23]. Adults usually perceive vaccination as an issue for children and/or pregnant women, therefore it is usually difficult to see adults who make themselves available for vaccination. It is therefore mandatory to develop adult-specific strategies to improve vaccination coverage among adults. Even though insufficient, the availability of PPE was an essential aspect of our best practices as no action could have been taking without the above. These findings are the same as in a similar study carried out in Ethiopia in 2020 on implementing nation-wide measles supplemental immunization activities in the context of COVID-19 [30].

One major lesson learned in this campaign was that intensifying communication activity several weeks before the implementation could improve the vaccination uptake and therefore help the government to reach its goal of addressing mortality due to cholera. This is in line with data a similar oral vaccination campaign carried out in Zanzibar in 2012. They recommended announcing the upcoming of a campaign a few months before the start and also reminding the target health areas frequently [31]. One major weakness of our study was its highly descriptive aspect and no major statistical analysis was carried out due to its objective which was to describe only what was achieved during the vaccination campaign. This is just an informative article and there is therefore limited reproducibility.

## Conclusion

The reactive mass vaccination campaign carried out in three regions of Cameroon in 2020 during COVID-19 pandemic was a success despite the high rate of refusal cases. This refusal could have been handled by a deep population sensitization right before the vaccination implementation starting with the central leaders, following with social mobilizers and then community leaders so that dissemination of information could start from there. COVID-19 on its own did not influence much vaccine hesitancy but it is insufficient sensitization that was the main factor fuelling vaccine refusal. COVID-19 did not negatively impact the outcome of the campaign as many vaccine-hesitant cases were handled with deep sensitization.

### What is known about this topic

- The case fatality rate of cholera in 2020 in Cameroon;
- The impact of mass vaccination campaign against cholera on reducing disease incidence.

### What this study adds

- Vaccination campaign against cholera coverage in 3 regions of Cameroon in 2020;
- Challenges of carrying out a vaccination campaign during COVID-19 pandemic;
- Best practices done during the vaccination against cholera campaign during COVID-19.
